# Formyl peptide receptor 2, as an important target for ligands triggering the inflammatory response regulation: a link to brain pathology

**DOI:** 10.1007/s43440-021-00271-x

**Published:** 2021-06-08

**Authors:** Kinga Tylek, Ewa Trojan, Magdalena Regulska, Enza Lacivita, Marcello Leopoldo, Agnieszka Basta-Kaim

**Affiliations:** 1 Laboratory of Immunoendocrinology, Department of Experimental Neuroendocrinology, Maj Institute of Pharmacology, Polish Academy of Sciences, 12 Smętna St, 31-343 Krakow, Poland; 2 Department of Pharmacy – Drug Sciences, University of Bari, via Orabona 4, 70125 Bari, Italy

**Keywords:** Formyl peptide receptors, Inflammation, Lipoxins, Resolvins, SPM’s (small pro-resolving mediators), Immune-related brain disorders

## Abstract

Formyl peptide receptors (FPRs) belong to the family of seven-transmembrane G protein-coupled receptors. Among them, FPR2 is a low affinity receptor for N-formyl peptides and is considered the most promiscuous member of FPRs. FPR2 is able to recognize a broad variety of endogenous or exogenous ligands, ranging from lipid to proteins and peptides, including non-formylated peptides. Due to this property FPR2 has the ability to modulate both pro- and anti-inflammatory response, depending on the nature of the bound agonist and on the different recognition sites of the receptor. Thus, FPR2 takes part not only in the proinflammatory response but also in the resolution of inflammation (RoI) processes. Recent data have indicated that the malfunction of RoI may be the background for some central nervous system (CNS) disorders. Therefore, much interest is focused on endogenous molecules called specialized pro-resolving mediators (SPMs), as well as on new synthetic FPR2 agonists, which kick-start the resolution of inflammation (RoI) and modulate its course. Here, we shed some light on the general characteristics of the FPR family in humans and in the experimental animals. Moreover, we present a guide to understanding the “double faced” action of FPR2 activation in the context of immune-related diseases of the CNS.

## Introduction

Formyl peptide receptors (FPRs) belong to the largest and functionally diverse family of 7 transmembrane chemoattractant G-protein-coupled receptors. FPRs are classified as Pathogen Recognition Receptors (PRRs) located on immune cells that play a key role in innate immunity due to their ability to recognize both, pathogen associated and damage-associated molecular patterns (PAMPs and DAMPs). In fact, FPRs were first identified on myeloid cell membrane, but subsequently their expression was demonstrated on neuronal, glial, endothelial and epithelial cells [1]. FPRs participate not only in host defense and regulation of inflammatory response but also in the migration, proliferation, superoxide production and in several physio-pathological processes due to their unique binding properties and interaction with structurally diverse ligands [2]. Actually, FPRs can interact with a wide range of compounds belonging to different chemical structures, from various endogenous peptides and proteins to non-peptide host-derived lipids and eicosanoids, but also covers many small-molecule ligands [3, 4].

Among FPRs, the FPR2 receptors are an attractive therapeutic target for researchers due to the functionality related to biased agonism and the diversity of bound ligands. In this review, we shed some light on the general characteristics of FPRs in humans and in experimental animals. Moreover, we present some of the crucial FPR2 ligands which may open opportunities for research in the context of immune-related diseases of the central nervous system including Alzheimer’s disease, depression and ischemia.

## Formyl peptide receptor family—an overview

The nomenclature of FPRs family is diverse, due to the fact that terminology of the same receptors was associated with the different manners of classification [5]. Therefore, to unify the terminology, the International Union of Basic and Clinical Pharmacology (IUPHAR) established a new lexicon based on the interaction of the receptor with the agonist. Based on these guidelines three members of FPRs family were identified in humans, namely FPR1, FPR2 and FPR3. On the other hand, despite the ordering of these nomenclature, the FPR2 and FPR3 receptors in the literature still often appear under other names, such as FPR1L and FPRL2 (these names refer to the common homology with other family members). Furthermore, the names ALX, FPR2/ALX, LXA4R are often used for the FPR2 receptor to refer to its interaction with the endogenous ligand A4 lipoxin (LXA4) [6, 7]. It is also worth to mention that in humans, at the beginning, the naming criterion for FPR1, FPR2 and FPR3 was based on the binding of formylated bacterial product formyl-methioninyle-leucyl-phenylalanine (*f*MLF), because the formyl receptor was first discovered as a target for this PAMP [8].

In humans all genes of the formyl receptor are located on chromosome 19. Moreover, they are characterized by high homology, e.g., the hFPR1 and hFPR2 receptors share sequence identity of 69%, hFPR1 and hFPR3 of 56%, while hFPR2 and hFPR3 about 83% (Table 1). Despite this sequence similarity, hFPR2 is more ubiquitous and was created as a result of gene amplification. According to the sequence analysis, hFPR3 is evolutionarily “the youngest” member of the FPR receptor family and seems to be more related to hFPR2 than to hFPR1, suggesting that it arose from gene duplication [9, 10]. They are all expressed on monocytes; in addition, hFPR1 and hFPR2 are also expressed on neutrophils [11] and hFPR1 and hFPR3 on dendritic cells (DC) [12, 13]. Formyl receptors, especially hFPR2, also maintain a relatively strong expression on cells of the nervous system including astrocytes and microglia [14].

**Table 1:** Formyl peptide receptors (FPRs) family names (IUPHAR-recommended and used previously)

IUPHAR-recommended FPR names	Other names (used previously)	Homology with FPR1	Homology with FPR2
FPR1	FPR, NFPR, FMLPR, FMLP	–	–
FPR2	FPR2/ALX, FPRH1, FPRL1, ALXR, RFP, LXA4R, FMLPX, HM63, FPR2A	69%	–
FPR3	FPRL2, FMLPY, FPRH2	56%	83%

Importantly formyl receptors also share overlapping functions. Originally, these receptors were thought to be only involved in neutrophil chemotaxis, but later discoveries have begun to highlight other functions, including: calcium efflux, clearance of infection, recruitment of immune cells, pro-resolving properties, but also a role as a background in the multiple diseases. The wide range of functions caused by the diversity of endogenous FPR ligands are not limited only to N-peptides [4]. FPR1 was for the very first time isolated from HL-60 cells that were differentiated into granulocytes [15] and prefer to bind short and flexible structures, such as *f*MLF for which they have a strong affinity [16]. The chimeric receptor approach showed that the affinity of FPR1 for *f*MLF was 400 times higher than that of FPR2 [17, 18].

Nevertheless, FPR2 is the only member of the formyl receptor family that interacts with all types of ligands, i.e., lipids, peptides, and proteins preferring mainly long, amphipathic peptides with a helix structure [16, 19]. To date, the evolutionarily youngest FPR3 is the least known member of the FPR family. Interestingly, only one peptide ligand with a high affinity for FPR3 is known [10, 11]. Furthermore, FPR3 receptor is highly phosphorylated, indicating that it rapidly internalizes after binding its ligands and thus may serve as a “decoy” receptor to restrict the binding of available ligands to other receptors [20]. Recently, some data have indicated a role of FPR3 in promoting calcium mobilization or chemotaxis [10, 11] but it certainly requires further research.

### Formyl peptide receptor family—animal species distribution

**Fig. 1:**
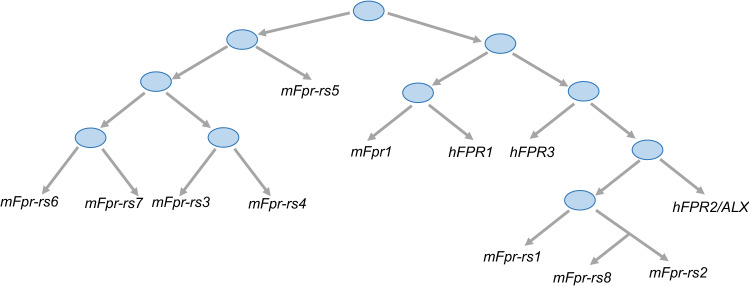
Homology between the human (h) and mouse (m) FPR family member genes. The “human group” contains three FPR proteins, the “mice group” includes eight FPR-related forms. *hFPR1* and *mFpr1* are in the same cluster, while *mFpr-rs1, mFpr-rs2* (called also *mFpr2*) and *mFpr-rs8* belong to the another cluster. They are closely related to hFPR2/ALX and hFPR3. Based on protein sequences, *mFpr-rs3, mFpr-rs4, mFpr-rs6, mFpr-rs7* and *mFpr-rs5* are closely related

The formyl receptor family has also become the focus of animal research. The presence of FPRs was found in guinea pigs, primates, rabbits, horses, rats, and mice, among others [5]. Considering that formyl receptors are present in a wide range of species, their structure, functionality, nomenclature, and homology with the human FPR family are very diverse. Currently, the most widely known formyl receptors in animals are those found in mice. The murine formyl receptor family includes 8 described formyl receptors: *mFpr1, mFpr2, mFpr-rs1, mFpr-rs3, mFpr-rs4, mFpr-rs6, mFpr-rs7, and mFpr-rs8* located on chromosome 17A3.2 [4] (Fig. 1). Scientific research has mainly targeted two direct orthologs between mouse and human with *mFpr1* and *mFpr2* represented by hFPR1 and hFPR2, respectively [21]. Although the human FPR family has murine orthologs whose high level of expression is also similar to that of humans on phagocytic leukocytes, the binding affinity for individual ligands is different. Literature data show a 100-fold lower affinity of *mFpr1* for *f*MLF and structural differences in the ligand binding domain. The mFpr1 receptor appears to be more similar to FPR2 in terms of its human ortholog [22, 23]. The structural differences between hFPR1 and *mFpr1* do not cover all aspects of functionality. Mice with the *mFpr1*^*−/−*^ phenotype revealed its strong association with host defense regulation. The targeted deletion of genes encoding *mFpr1* but also *mFpr2* seems to confirm these results. Animals with that deletion show reduced resistance to bacterial infections; however, the fertility and viability of the animals are not affected [24, 25]. The *mFpr2* and *mFpr-rs1* receptors show high homology to human FPR2 and FPR3. Moreover, the knockout study mice *mFpr2*^*−/−*^ revealed the possibility of a functional crossover between hFPR3, hFPR2, and *mFpr2,* respectively [26, 27]. Studies based on these animals have also established that despite mFpr2 has a low affinity for *f*MLF, it binds with high affinity to several peptide agonists that activate human FPR2/ALX, including the amyloidogenic proteins serum amyloid A [28–31] and amyloid β _(1–42)_. Mouse Fpr2 is also a receptor for F2L (which is also a strong agonist of hFPR3). These findings indicate that mouse Fpr2 share pharmacological properties with human FPR2/ALX. It is very important in the context of the result of studies conducted in FPRs-deficient mice, indicating their translational potential.

The remaining members of the formyl receptor family do not seem to exhibit as many distinctive features. It may be related to the complex evolution of genes and sequence divergence between orthologs. Among the genes encoding *mFpr,* there is the pseudogene *mFpr-rs5 (ψmFpr-rs3)* which does not encode a functional receptor, but it does not possess the features characteristic of pseudogene [4, 21]. *mFpr-rs1, mFpr-rs3, mFpr-rs4, mFpr-rs6, and mFpr-rs7* represent chemosensory vomeronasal GPCR receptors [32, 33]. The biological function of *mFpr-rs1* is still unclear. Although *mFpr-rs1* overlaps many functions and structural features with hFPR2 its ability to activate the human and mice ligands is very low. The underlined data demonstrate the commonality of many structural and pharmacological features of both human and mice members of the FPR family.

## Conformational changes and biased agonism of FPRs

According to the literature, the FPR family is a group of G-protein-coupled receptors and belongs to one of the most diverse groups of receptors, namely: 7 transmembrane receptors (7 TM) [34, 35]. In general, the FPR family receptors consists of a few conservative elements: the extracellular N-terminus, seven transmembrane domains (TM1–7) connected via three intracellular and extracellular loops (IL1–3, EL1–3) and the intracellular C-terminus. Furthermore, in some receptors, there is an extra eighth helix in the polypeptide chain that is parallel to the inner surface of the cell membrane [36, 37]. The extracellular domains (EL1–3) are responsible for the detection of ligands and their access to the structural core, while intracellular domains (IL1–3) bind to a variety of cytoplasmatic systems, such as G proteins, arrestin or receptor kinases coupled with G proteins [38]. Transmembrane TM1–7 helixes participate in binding and signal transmission into the cell through conformational changes that are essential for receptor activity [39]. Two highly conserved motifs are directly involved in the conformational changes: NPXXY in TM7, which is responsible for activating the receptor and E/DRY (combining TM3 and TM6), which acts as a specific "ion blocker" that maintains the stabilization of the receptor conformation [40, 41].

It is intriguing to observe that among formyl peptide receptors, FPR2 have properties to functional changes, which depend on this receptor conformation. Emerging data suggest that FPRs form higher order structures (e.g., FPR1/FPR2 heterodimers, FPR2 homodimers, FPR1 homodimers), which leads to altering the downstream intracellular signaling pathways by allowing colocalization of effector domains, enhancing intracellular activation, or creating new ligand specificity [42, 43]. Cooray et al. have indicated that FPR2 homodimers and FPR2–FPR1 heterodimers occur constitutively in leukocytes and alters the activation of signaling pathways in response to specific ligands [44]. Peptide ligands also play a role in dimerization: annexin A1 (ANXA1) and LXA4 promote FPR2 homodimerization, while peptide Ac2–26 stimulates FPR2–FPR1 heterodimerization. Interestingly, FPRs also form oligomers with scavenger MARCO receptors (macrophage receptor with collagenous structure). Interactions between FPR and MARCO receptors have been demonstrated by bioluminescence and co-immunoprecipitation studies and fulfill their functions in agonist-evoked changes in cyclic adenosine monophosphate (cAMP) levels and extracellular signal-regulated kinases (ERK1/2) phosphorylation, as well as signal transduction in glial cells via Aβ1–42 [45]. Importantly, the FPR2 conformational changes (ligand-dependent) determines its action [46].

**Fig. 2:**
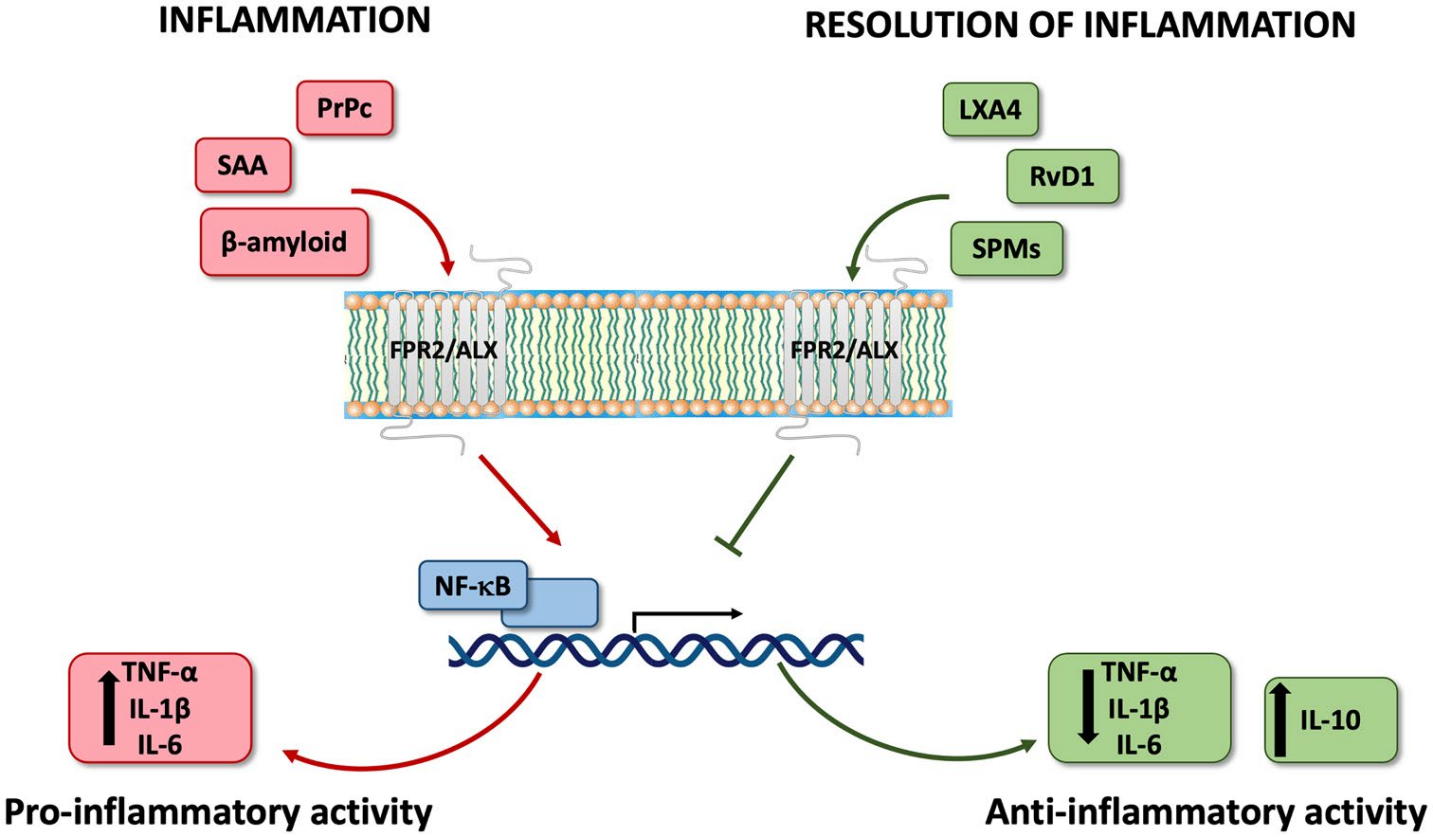
Ligand-biased signaling via FPR2 leads to dual effects: pro-inflammatory on the one hand and pro-resolving on the other. A variety of endogenous ligands exert pro-inflammatory (*SAA* Serum Amyloid A, *PrPc* Prion Protein, *ANXA1* Annexin A1) and pro-resolving (*LXA4* Lipoxin A4, *RvD1* Resolvin D1, *SPMs* Specialized Pro-resolving Mediators) effects. SAA, PrPc, amyloid-β elicit proinflammatory signals and stimulate the release of pro-inflammatory cytokines (e.g., TNF-α, IL-1β, IL-6). This FPR2 signaling is counteracted by pro-resolving agonists that suppress the expression of pro-inflammatory cytokines and increase the release of anti-inflammatory factors (e.g., IL-10). (Image generated by Biorender)

On the other hand, protein and lipid ligands bind to different FPR2 binding sites (Fig. 2). Lipoxins A4 (LXA4) bind at 7TM and 3rd extracellular loop, while peptide ligands, such as ANXA1 or serum amyloid A (SAA), bind at the NH2-terminal domain or 1st two extracellular loops [47, 48].

After binding of the ligand, FPR2 is activated and triggers several agonist-dependent signal transduction pathways through the involvement of the Gαi1, Gαi2 and Gαi3 G-receptor subunits [5]. In fact, the effects observed after FPR2 activation include the activation of phospholipase A2 (PLA2), phospholipase C (PLC) isoforms, protein kinase C (PKC), phosphoinositide 3-kinase (PI3K), protein kinase B (Akt), mitogen-activated protein kinase (MAPK) pathway as well as p38MAPK, which modulate proliferation, differentiation, apoptosis, cellular communication and other intracellular functions. Furthermore, phosphorylation of cytosolic tyrosine kinases, phosphorylation and nuclear translocation of regulatory transcription factors, calcium release and oxidant production so far were demonstrated [49]. Among the post-translational modifications of FPR2, phosphorylation processes, which are determined by a balance between protein kinases and protein phosphatases, seem to be of great importance [1]. Therefore, despite the fact that protein phosphorylation is limited to specific phospho-sites, and it is not the only post-translational change (which also include methylation, acetylation, sumoylation or ubiquitination) occurring after FPR activation by agonists, understanding the mechanisms of their regulation may be crucial for the development of new pharmacotherapy of CNS diseases. Ligand-dependent stimulation of G-protein-coupled receptors may also lead to transactivation process, which activates signaling from receptors tyrosine kinase (RTK) [50]. Among others, TrKA receptor activation results in phospohorylation of various tyrosine residues (e.g., Y490, Y751 or Y785). Phosphorylated tyrosine residues form docking sites for other proteins and trigger the activation of Ras/MAPK, PI3K/Akt as well as PLC γ/PKC pathways [51]. Interestingly, several features of TrkA receptor transactivation are noteworthy and differ significantly from other transactivation events, first of all, because it is slower. However, given the role of the mentioned signaling cascades in the physiological and pathological processes in the brain and in the action of CNS-active drugs, the TrkA transactivation by FPRs agonists may provide an innovative strategy for the treatment schizophrenia, depression and other mental illnesses.

At the same time, it should be strongly emphasized that the FPR2 downstream signaling pathway activation, depends not only on the chemical structure of the ligand but also on the cell type involved [14, 52], which is important in understanding how FPR2 activation elicits different cellular responses leading to inflammation and its resolution. For example, SAA binding increases the expression of the NF-кB, whereas LXA4 suppresses NF-кB activity [46]. Therefore, FPR2 enables the switch of both pro-inflammatory action to pro-resolving because of the diversity of intracellular signaling cascades from GPCR activation.

In 2019, Raabe et al. discussed the biased perspectives on FPRs. According to those authors, the “classic” view about ligand/receptor interactions accounts only for agonists (which leads to activation) and antagonists (inhibit the activation) [53, 54]. FPR2–ligand interactions lead to totally different cellular responses, a finding, which completely questions this classical concept of receptor–ligand interaction. This phenomenon—biased agonism—explains how different FPR2 agonists do not lead to the same effects and why FPR2 agonists play essential roles in the control of active inflammation resolution and host defense. What is more, FPR2 is unusual, because it can switch from a pro-inflammatory to anti-inflammatory response while at the same time maintaining the former at a low but possibly life-saving level [55].

## Inflammatory response and FPR2 ligands

The inflammatory response is one of the main process in the organism. Among the processes of inflammatory origin, acute inflammation is a protective, self-limiting process that disappears after the removal of the insult in the absence of major damage to the body. Several phases of inflammation, including initiation, propagation, and resolution, have been demonstrated. Recently it has been suggested that these phases do not develop sequentially but rather overlap [56]. The physiological outcome of the acute inflammatory response is the restoration of tissue homeostasis and functionality, culminating in tissue repair, and is followed by the resolution phase [57, 58]. However, when the mechanisms controlling this complex reaction, triggered by several factors including proteins, lipids and stimulatory signals derived from injured cells or by inflammatory mediators (e.g., chemokines, cytokines) fail, an uncontrolled inflammatory reaction can be detrimental, which indeed is a driving pathogenetic mechanism for a wide range of immune-related diseases.

There is a lot of evidence that in the course of the prolonged inflammatory response and neurodegeneration, non-formyl peptide FPRs agonists are involved. This group of ligands activates FPRs independently from the presence of an *N*-formyl group, showing a particular preference for the interaction and activation of FPR2.

Undoubtedly, the serum acute-phase protein (SAA) is of particular importance among them because of an unfavorable role in chronic inflammation and amyloidosis. The pro-inflammatory effects of SAA caused by stimulation of FPR2 in phagocytes, epithelial cells and T lymphocytes, lead to the production of inflammatory mediators [43, 59–61]. Interestingly, some data postulated that native SAA may exhibit cytokine-like properties but whether this effect is related to FPR2 activation still remains unclear and is a subject of scientific debate [62, 63].

Among the amyloidogenic agonists of FPR2 the cellular prion protein fragment (PrP^c^), a glycoprotein highly expressed in the brain is highlighted. The role of PrP^C^ in amyloid β (Aβ) oligomer‐induced synaptic impairment is of great interest [64]. In fact, impairment of LTP by Aβ oligomers isolated from the brains of AD patients was attenuated by pretreatment with an anti‐PrP^C^ antibody [65, 66]. Moreover, some data pointed out the role of PrP^C^ in synaptotoxicity mediated by soluble Aβ. On the other hand, in some studies the effects of PrP^C^ in the LTP alterations and memory deficits in mouse models of AD were not seen [67, 68]. PrP^C^ fragment, through its interaction with FPR2 in glial cells, induces calcium mobilization, enhances chemotaxis (e.g., via MCP-1) and leads to potentiation of the inflammatory response. Among the cytokines released from glia cells in response to PrP^C^, there are: TNF-α, IL‐1β, IFN‐γ, or IL‐6, which reportedly accelerate AD progression in both AD patients and in the animal model of AD [69, 70].

In addition to SAA and PrP^c^, two other amyloidogenic peptides have also been described: 42-amino acid form of Aβ amyloid peptide (Aβ42) and humanin, which exert an agonistic effect on FPR2. Despite the fact that both peptides, by activating FPR2, induce migration and increase the phagocytic activity of monocytes in the brain, they have a different role in the course of Alzheimer's disease. Aβ42 is involved in the fibrillary tangle formation and deposition in the brain of AD patients [71, 72]. Moreover, via interaction of microglial cells with FPR2 Aβ42 increase the inflammatory cytokines production, including TNF-α, interleukins (IL‐1β, IL‐6), interferon‐γ, and chemokines, such as CCL2, CXCL8, CXCL10 and CCL3 [73, 74].

In contrast, the already mentioned humanin has the opposite, i.e., neuroprotective activity [75]. In fact, humanin, by inhibiting Aβ interaction with FPR2 in phagocytes, probably reduces aggregation and generation of fibrillary formations. Perhaps also the ability of humanin to interact with other FPRs, e.g., FPR3 [75, 76] play a crucial role in these phenomena.

The “dual-faced” FPR2 agonists include annexin A1 (ANXA1) and its bioactive N-terminus domains (Ac2–26 and Ac9–25). ANXA1 is a glucocorticoid-regulated phospholipid-binding protein of 37 kDa, expressed in a variety of cell types. It seems that the dual properties manifested by ANXA1 are mediated by peptides derived from its N-terminus domain (Ac2–26 and Ac9–25), which are presumably generated at sites of inflammation. Interestingly, at high concentration the ANXA1 peptides fully activate FPR1, just as the conventional agonists and induce pro-inflammatory response. On the other hand, at low concentrations they only demonstrate a partial activity at FPR1, leading to the inhibition of adhesion and transmigration of leukocytes, reducing the intensity and duration of the inflammatory response while intensifying proliferation and invasion of epithelial cells [77]. Moreover, it is suggested that both (Ac2–26 and Ac9–25) peptides use FPR2 for their anti-inflammatory actions [78], but there are also data postulating that other receptors, including FPR3, are involved in these pro-resolving effects [79].

Moreover, the role of ANXA1 in the behavioral disturbances, such as anxiety is widely discussed. In fact, the absence of ANXA1 protein even more than the absence of its main receptor (namely FPR2/3) is indispensable to the suppressive action of glucocorticoids on the HPA axis, as well as to the hippocampal homeostasis by preventing neuronal damage in the course of depression [80]. On the other hand, in FPR2/3-deficient mice data showed a behavioral disinhibition and reduced anxiety [81], manifested by the increased climbing exploratory activity in an open-field test, as well as superior performance on a novel object recognition test, just to mention a few. These effects were accompanied by an increase in blood plasma corticosterone, which does not exclude the possibility of a compensatory effect and/or changes in ANXA1 level. This issue undoubtedly requires further detailed studies. Nonetheless, the crucial role of FPR2 receptors in mediating the behavioral deficits at the cognitive–emotional interface are clearly confirmed by the Boc-2 administration to wild-type mice, which followed the deficits observed in the above-mentioned FPR2/3-deficient mice [81].

Recently, data have demonstrated that in the brain, ANXA1 is engaged in the regulation of the blood–brain barrier (BBB) integrity of patients with multiple sclerosis [82]. Furthermore, ANXA1 may be involved in the occurrence and progression of acute severe traumatic brain injury [83]. Moreover, Wang et al. found that the expression of ANXA1 decreased after cerebral hemorrhage, and the increase in the expression of ANXA1 could alleviate neuronal necrosis, and reduce brain edema after cerebral hemorrhage [84]. Interestingly, Luo et al. found that ANXA1 could also exert neuroprotective effects on brain damage by polarizing microglia cells into M2 phenotypes [85].

ANXA1 was reported to also be associated with the early stage of AD in patients and in animal models. By inhibiting the secretion of inflammatory mediators stimulated by Aβ, ANXA1 could stimulate microglial phagocytosis of Aβ and reduce the level of Aβ [86]. In fact, some data show that ANXA1 expression is reduced in AD patients, which may be related to an increased degree of neurodegeneration. The decreased expression of ANXA1 in patients with mild cognitive impairment and AD might contribute to the increased neuroinflammation and cognitive deficits [87].

## FPR2 agonists in the course of the resolution of inflammation

The correct flow of the resolution of inflammation (RoI), which is an active process, requires proper endogenous activation that induces a switch from the release of proinflammatory molecules to the secretion of pro-resolving mediators. In this event, the so-called specialized pro-resolving lipid mediators (SPMs) play a prominent role, because they modulate leukocyte infiltration and activities, as well as anti-inflammatory cytokine release to terminate inflammation [88]. These molecules, including lipoxin A4 (LXA4), derived from arachidonic acid (AA), and the D-series resolvins (RvD1) derived from docosahexaenoic acid (DHA) are key paracrine and autocrine biochemical signaling molecules in the CNS. They are reported to be involved not only in the RoI by triggering the processes that reduce the expression of pro-inflammatory response, but also, in the case of RoI deficits, in the progression of neurodegenerative and neuropsychiatric diseases [89, 90]. In fact, SPMs activate cascades that induce remodeling within sites damaged by inflammatory processes. Most of the effects of RoI are mediated through FPR2, which is able to promote several processes crucial for resolution of inflammation, including neutrophil extravasation blockade, promotion non-phlogistic monocyte recruitment, suppression of proinflammatory mediators while potentiating anti-inflammatory cytokines release and macrophage phagocytosis and efferocytosis, altering macrophages phenotype and instructing cells to favor repair [91, 92]. Interestingly, the anti-inflammatory effects rely mostly on suppressive action, while pro-resolving effects are mediated by the activation of specific inherent processes; however, the RoI is the final result of both [91, 93]. Recently, it has been found that SPMs elicit “mild to moderate effects”, which, led to the balance between proinflammatory and anti-inflammatory reactions [91]. It should be mentioned, that in the brain the course of inflammatory response is slightly different due to the collective interaction of various brain cells (microglia, astrocytes, oligodendrocytes, and NG2 glia) and, in some cases, peripheral immune cells. Therefore, a great deal of importance is given to SPMs which can act on both glia and neurons [93] and they include lipoxins and resolvins.

### Lipoxins

Lipoxins have emerged as prominent chemical mediators whose synthesis is switched on during an inflammatory response, which allows the RoI. In classical lipoxin biosynthesis in leukocytes and epithelial cells, arachidonic acid undergoes double, transcellular oxidation catalyzed by lipoxygenases (LOX), resulting in the formation of two derivatives of lipoxin A (LXA4) and lipoxin B (LXB4) [93]. On the other hand, in the second pathway of lipoxin synthesis, aspirin-dependent lipoxin epimers: AT-LXA4 and AT-LXB4 are formed under the influence of acetylated cyclooxygenase (ASA-COX2). Lipoxin A4 (LXA4) and its AT-LXA4 epimer act primarily through the FPR2 receptor [94]. In addition, LXA4 can activate other receptors, such as an orphan G-protein-coupled receptor (GPR32), aryl hydrocarbon receptor, estrogen receptor and high affinity cysteinyl leukotriene receptor [95–97].

**Fig. 3:**
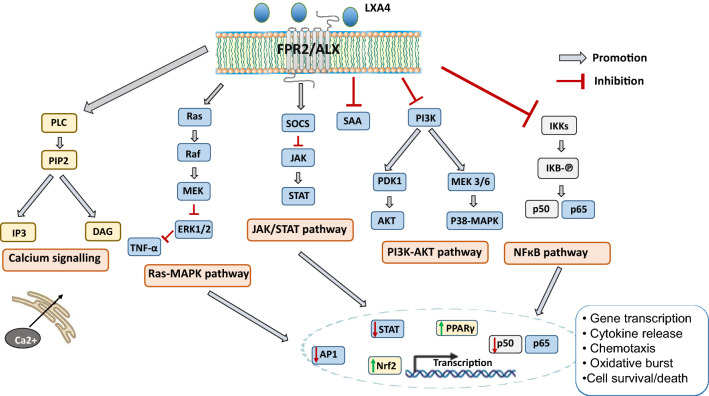
Binding of LXA4 to FPR2 receptor results in the activation of many intracellular signaling pathways. Depending on the cell type, LXA4 have different effects on PI3K/AKT signaling pathway. In macrophages, lipoxins have an anti-inflammatory effect through the activation of PI3K/AKT pathways which leads to an increase in their life span. By increasing cytosolic calcium levels, LXA4 is involved in the recruitment of neutrophils at the site of inflammation. LXA4 controls the synthesis of pro-inflammatory cytokines by inhibiting the activation of the NF-κB and by increasing the SOCS mRNA level. By inhibiting the activation of transcription factors including NFkB and AP-1, LXA4 up-regulates the levels of Nrf2 and PPARγ—factors which suppress the expression of pro-inflammatory genes. *LXA4* Lipoxin A4, *NF-κB* nuclear factor-κB, *SOCS* cytokine signaling suppressors, *AP-1* activator protein 1, *Nrf-2* nuclear factor erythroid 2-related factor 2, *PPARγ* peroxisome proliferator-activated receptor gamma

Binding of LXA4 to FPR2 receptor results in the activation of many intracellular signaling pathways. Simultaneously, the conformational changes following the attachment of LXA4 prevents binding of other ligands, e.g., amyloid β or SAA to the FPR2 [19]. Among signaling cascades, the cell-dependent activation of the PI3K/AKT pathway by LXA4 is of key interest [98]. LXA4-mediated modulation of the neutrophil recruitment to the site of inflammation by increasing cytosolic calcium levels is important in the resolution of inflammation [99]. Moreover, LXA4 anti-inflammatory effect is also associated with the inhibition of the NF-κB (nuclear factor-κB), which in turn, leads to a reduction in the transcription of pro-inflammatory cytokines. Simultaneously, LXA4 increases the level of mRNA for cytokine signaling suppressors (SOCS). On the other hand, LXA4, by inhibiting the activation of transcription factors including NFκB and AP-1 (Activator protein 1) [100], up-regulates the levels of nuclear factor erythroid 2-related factor 2 (Nrf2) and peroxisome proliferator-activated receptor gamma (PPARγ), which are the factors which suppress the expression of pro-inflammatory genes [98] (Fig. 3). Resolving the inflammation and restoring LXA4 signaling has been shown to reduce the severity of Alzheimer’s disease such as neuropathology including the decrease in amyloid plaques, tau phospohorylation and inflammation as well as leading to the improvement in the cognitive performance in the 3xTg-AD mouse model [101]. Moreover, the combined administration of LXA4 and resolving E1 terminated inflammation in a murine model of AD [102]. The mechanism of LXA4 and AT-LXA4 has not been defined unequivocally, nevertheless it is postulated that both agonists reduce the secretion of pro-inflammatory mediators, such as TNFα, while LXA4 has also been shown to promote the release of anti-inflammatory factors and to exhibit the ability to reduce Aβ and phosphorylated tau levels [89].

### Resolvins

Resolvins are the second important class of FPR2 agonists that play an important role in the positive regulation of inflammatory processes. They are a group of compounds, derivatives of docosahexaenoic acid (DHA)—resolvin D and eicosapentaenoic acid (EPA)—resolvin E. The formation of resolvins is the result of the process taking place at the final stage of acute inflammation as a result of the interaction of cells, i.e., neutrophils, macrophages, platelets or endothelial cells (transcellular biosynthesis). The synthesis of D-series resolvins from docosahexaenoic acid (DHA) is catalyzed by lipoxygenase (15-LOX) or acetylated aspirin cyclooxygenase-2 (COX-2). The initially formed 17R-hydroperoxydocosahexaenoic acid (17R-HDHA) is transformed by epoxidation and with the participation of 5-LOX into D resolvins 1 to 4, which differ in the stereochemical asymmetry of the carbon chain. In parallel, the transformation of DHA under the influence of ASA-COX-2 leads to the formation of AT-RvD1 to 4 [103]. The synthesis of E-series resolvins occurs by conversion of eicosapentaenoic acid (EPA) catalyzed by ASA-COX-2 and 5-LOX leads to resolvin E1 (RvE1) and resolvin E2 (RvE2) formation.

**Fig. 4:**
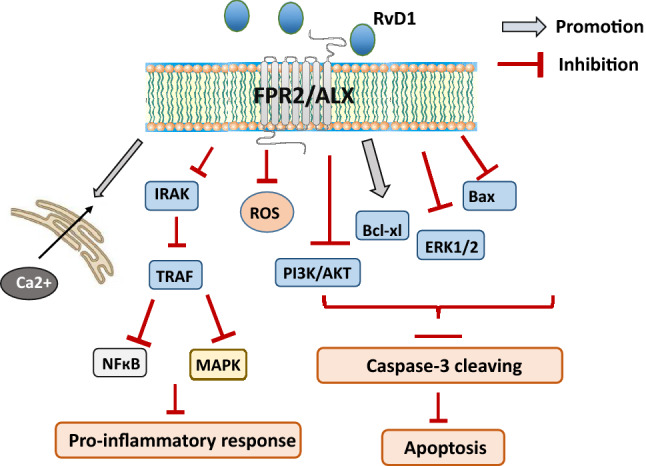
After binding to FPR2, RvD1 can promote cell survival by calcium release, Erk1/2 and PI3K/Akt signaling activation or blocking the TNF-α signaling as well as caspase-3 activity. RvD1 could also promote bcl-xL expression, leading to cell survival. Interaction with FPR2 negatively regulates downstream IRAK1/TRAF6/NF-κB or MAPKs signaling pathways. *RvD1* Resolvin D1, *NF-κB* nuclear factor-κB, *TNF-α* tumor necrosis factor α

RvD1 interacts with the GPCR-32 receptor as a potent agonist to signal for pro-resolving responses but can also directly activate FPR2 with a high affinity [77]. Numerous studies have shown that resolvins inhibit the migration of inflammatory cells, stimulate macrophages to phagocytosis of apoptotic neutrophils, inhibit NF-κB activation and secretion of proinflammatory cytokines, thereby contributing to the suppression of inflammatory processes [104]. Moreover, RvD1 can promote cell survival by calcium release, Erk1/2 and PI3K/Akt signaling activation or blocking the TNF-α signaling as well as caspase-3 activity. Furthermore, RvD1 could promote bcl-xL expression, Interaction with FPR2 negatively regulates downstream IRAK1/TRAF6/NF-κB or MAPKs signaling pathways [80, 105]. All of the above data indicate that RvD1 may modulate microglial pro-inflammatory polarization and may play an important role in the resolution of inflammation (Fig. 4).

In line, in PC12 cell cultures the beneficial impact of RvD1 on the IL-4 induced expression of alternative microglia stimulation markers was observed. This anti-inflammatory and pro-resolving effects of RvD1 was related to the activation of STAT6 and PPAR-γ signaling pathways [106]. In addition, it was found that an increase in the production of D1 resolvin may be one of the mechanisms protecting the cells against ischemic injury, resulting in the protective effect on CA1 neurons of the hippocampus and cognitive functions. This action of RvD1 is probably also related to its modulatory impact on the PPAR-γ pathway [85].

Some data postulate a possible therapeutic potential of RvD1 in Parkinson’s disease. It is based on the observation from the PC12 cultures, where RvD1 dose-dependently inhibited MPP + induced upregulation of cell apoptosis and cellular damage evoked by TNF-α and IL-6 production via suppression and ERK and p-38 pathways [107]. In addition, in an in vivo model of Parkinson's disease in rats induced by 30-day LPS administration, the combined treatment with RvD1 and RvD2 prevented the development of behavioral deficits and the activation of the TLR4/NF-κB pathway [108].

Nevertheless, much more data points to the antidepressant potential of RvD1 in many experimental models. For instance, it was described, that in animal models of depression, some resolvins counteracted the depressive-like behavior. In fact, intraventricular administration of RvD1 or RvD2 attenuated the LPS-induced depression-like behaviors in the tail suspension test (TST) and forced swim test (FST) in murine chronic unpredictable stress (CUS) model, which may indicate an antidepressant effect of RvD1 and RvD2 [109]. Also, in studies using the murine model of depression, RvD1 has been shown to have an antidepressant effect, strongly dependent on the activation of FPR2 and in consequence, on MAP/ERK, PI3K/Akt but also AMPA signaling [110]. Importantly, in the mouse model of fibromyalgia-associated depression, intravenous RvD1 and RvD2 administration increased dopamine and glutamine cortical levels and limited the deficiencies of serotonin, suggesting the positive effect on neurotransmitter imbalance in depression [111]. Simultaneously some clinical studies suggest that RvD1 may be an attractive marker in manic, depressive and euthymic states of bipolar disorders. In fact, the levels of RvD1 were enhanced in manic and depressive states in comparison with the appropriate control groups [112]. Since RvD1 level correlated with an increase in the c-reactive protein, it is possible that RvD1 concentration should also be indicative of the presence of a subclinical inflammation, especially in the course of acute episodes to compensate for the inflammatory response. The usefulness of RvD1 as an indicator of the anti-inflammatory process has been confirmed by a positive correlation between RvD1 and neutrophil count. Thus, the assessment of RvD1 may be a new potential marker in studies of psychiatric disorders associated with inflammatory processes.

Various reports postulate that also RvD1 of the AT-RvD1 series, which was formed as a result of the action of ASA-COX2, exerts anti-inflammatory and pro-resolving effects and is many times more stable that LXA4 and RvD1. In fact, the data from in vitro and in vivo studies show that the peripheral administration of AT-RvD1 prevented astrogliosis and improved short- and long-term potentiation (LTP) enhancement of the hippocampus in mice [113]. Furthermore, improvement in the sensorimotor function and memory after traumatic brain injury (TBI) in mice leads to the conclusion that the reduction of long-term inflammation limits the decline in neurological function [114]. Simultaneously, beneficial responses were observed after intravenous administration of AT-RvD1 expressed as increased levels of cortical dopamine and glutamate and reduced depletion of serotonin in a mouse model of depression associated with fibromyalgia, which suggests that AT-RvD1 activity normalizes neurotransmitters levels in depression [111].

### Synthetic FPR2 agonists

Lipoxins and resolvins exert strong endogenous anti-inflammatory effects but their chemical and metabolic liability [115] greatly hamper their development as potential pro-resolving drugs. In fact, LXA4 is subject to metabolism by prostaglandin dehydrogenase at C_15_ and ω-oxidation at C_20_. Therefore, there has been and still, there is a great interest to develop lipoxin analogs less susceptible to metabolic deactivation with a longer biological half-life [116, 117].

**Fig. 5:**
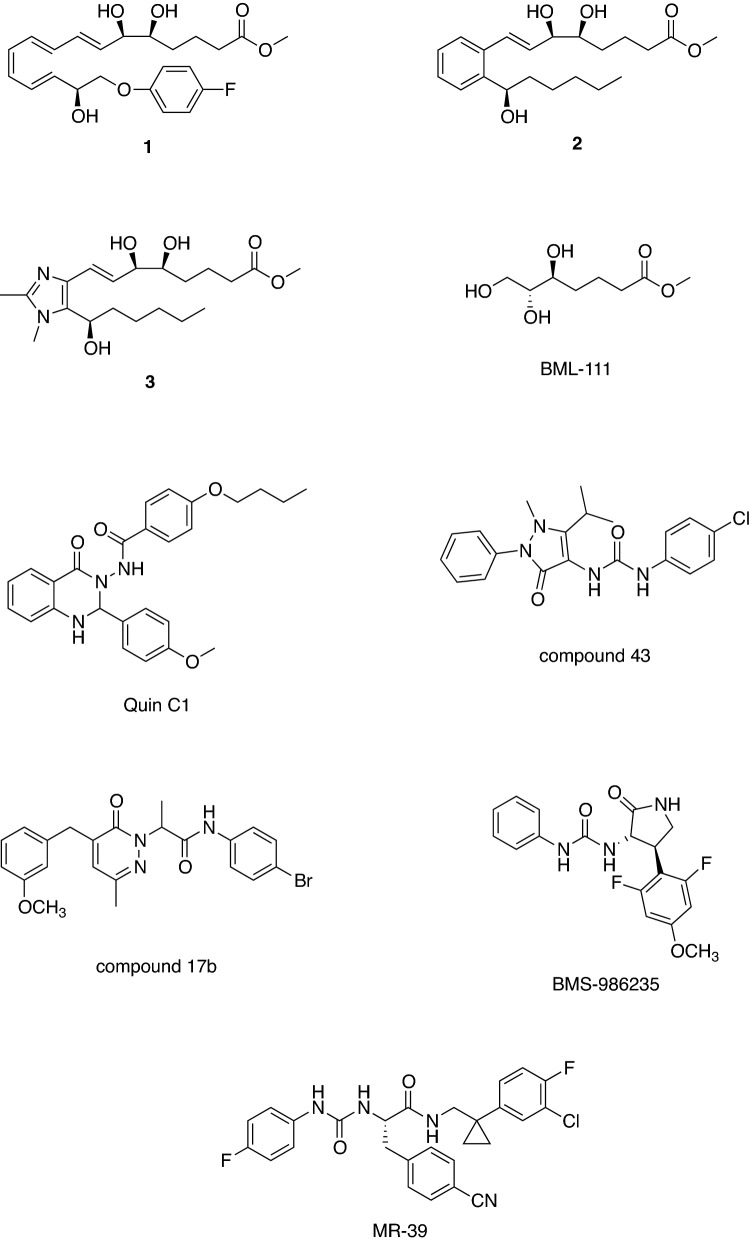
Structures of the lipoxin mimetics and small-molecule Formyl peptide receptor 2 (FPR2) agonists

The first generation of lipoxin analogs was designed to enhance biostability at C_15_ and the ω-end. For example, compound **1** (Fig. 5) was able to inhibit the transmigration of human neutrophils at a dose range comparable to LXA4 [22]. However, the therapeutic potential of these analogs was limited due to rapid in vivo clearance after oral or intravenous administration.

The second generation of lipoxin mimetics featured a benzene ring to replace the triene system of LXA4 (the so-called benzo-LXA4), exemplified by compound **2** (Fig. 5), which demonstrated potent potential therapeutic in several models of peripheral inflammation [44, 45].

The high lipophilicity of the second generation of lipoxin mimetics led to the development of the less lipophilic third generation in which the benzene ring was replaced with heteroaromatic rings (imidazole, oxazole). These compounds, exemplified by compound **3** (Fig. 5), showed in vitro anti-inflammatory activity being able to attenuate LPS-induced NF-κB activity with a potency similar to LXA4 [46, 47] and reduced the inflammatory process in vivo in a model of zymosan-induced peritonitis. None of the lipoxin mimetics has been tested in animal models of neurodegenerative diseases and, thus, there are data about their ability to cross the blood–brain barrier and to accumulate into the brain.

Besides lipoxin mimetics, several small-molecule FPR2 agonists with promising therapeutic potential have been developed from both pharmaceutical companies and academia. The FPR2 agonist BML-111 (Fig. 5) is able to reduce inflammation and neutrophil infiltration and to potentiate the release of anti-inflammatory factors (e.g., IL-4, IL-10) in various inflammatory-based disorders [106, 116, 118–120]. A recent study demonstrated the efficacy of BML-111 in the cerebral ischemia–reperfusion injury in rats (Fig. 2) [94]. In the ischemic brain treatment LXA(4)ME suppressed neutrophils infiltration and lipid peroxidation levels; inhibited the activation of microglia and astrocytes, reduced the expression of pro-inflammatory cytokines (e.g., TNF-α and IL-1β), while up-regulated the expression of anti-inflammatory cytokines (e.g., IL-10 and TGF-β1). Interestingly, the activation of NF-κB was also inhibited by LXA(4)ME, which suggested that LXA(4)ME afforded a strong neuroprotective effect against cerebral ischemia–reperfusion injury, and that these effects might be associated with its anti-inflammatory property [121].

Among the small-molecule FPR2 agonists, the quinazolinone derivative Quin-C1 (Fig. 5) is a potent agonist as it induces FPR2-mediated intracellular Ca^2+^ mobilization in the nanomolar range. Quin-C1 showed anti-inflammatory properties in a mouse model of bleomycin-induced lung injury being able to decrease the expression of IL-1β and TNF-α [122]. Another small-molecule FPR2 agonist, which is also an FPR1 agonist, is the chloropyrazolone derivative “Compound 43” (Fig. 5). This compound is able to mobilize intracellular Ca^2+^ and inhibit PMN migration stimulated by IL-8 and *f*MLF [123]. In a recent study, the intracellular signalling pathways activated by Compound 43 and by the pyridazin-3(2*H*)-one FPR2 agonist known as “compound 17b” have been comparatively studied evidencing biased-agonist properties for the two compounds. In CHO cell overexpressing FPR2 and in primary cardiomyocites “compound 17b” showed a marked biased effect as it induced ERK1/2 and Akt1/2/3 phosphorylation along with 30-fold bias away from intracellular Ca^2+^ mobilization relative to “compound 43”. In addition, “compound 17b” reduced necrosis in isolated cardiomyocytes and inhibited the release of pro-inflammatory IL-1β after stimulation with TGF-β [124].

The pyrrolidinone FPR2 agonist BMS-986235 (Fig. 5), recently disclosed by Bristol-Meyer Squibb, shows high potency and selectivity for FPR2 and is able to inhibit neutrophil chemotaxis and stimulate macrophage phagocytosis in cellular assays. BMS-986235 is also able to improve cardiac function in a mouse model of heart failure [125].

We have contributed to the field of FPR2 agonists by developing a series of ureidopropanamide-based agonists [126, 127] that has its origin from the gastrin-releasing peptide receptor antagonist PD-175266 and the neuromedin B receptor antagonist PD-168368, both potent FPR1/FPR2 agonists. A medicinal chemistry campaign led to the identification of the selective FPR2 agonist MR39 (Fig. 5) [126] that shows favorable pharmacokinetic properties. In fact, MR39 is stable to oxidative metabolism in rat liver microsomes (t_1/2_ = 48 min) and shows good passive permeability through an hCMEC/D3 cells monolayer, an in vitro model of the blood–brain barrier. MR39 demonstrated protective and anti-inflammatory properties as it lowered IL-1β and TNF-α levels in LPS-stimulated primary rat microglia cell cultures [126]. Moreover, MR39 and related analogs exerted neuroprotective effects in LPS-stimulated rat primary microglial cells at dose ranges comparable to LXA4 but lasting longer (unpublished data). MR39 provided promising results also in relation to the shift to the alternative microglia activation and the synthesis of anti-inflammatory cytokines. Thus, MR39 and its analogs are prospective tools to study the therapeutic potential of FPR2 agonists in the pharmacotherapy of CNS diseases [127]. It worth noting that the wide chemical diversity of FPR2 agonists might imply biased FPR2 signaling. Therefore, a detailed pharmacological analysis of existing FPR2 agonists will provide valuable pieces of information in the search of FPR2 agonists effective in the resolution of inflammation.

## Conclusions

The FPR2 is a versatile transmembrane protein belonging to the class of G-protein-coupled receptor family. FPR2 recognize various ligands with significantly different structures, such as non-formyl peptides, endogenous peptides, structurally unrelated lipids as well as synthetic small pro-resolving molecules. Therefore, FPR2s is highly “promiscuous” in terms of ligand recognition, which means that it can be activated by agonists with pro-inflammatory as well as pro-resolving properties. This creates a unique opportunity for switching from pro- to anti-inflammatory profile of FPR2 activation. This is of utmost importance for the treatment of various chronic CNS inflammatory-related diseases, since traditional anti-inflammatory therapies only reduce the mounting of the inflammatory response but also impair some relevant mechanisms that trigger the resolution phase. Therefore, a novel and innovative approach to modulating the inflammatory response is needed. Opportunities are given by SPMs, which in addition to their well-recognized role as modulators of inflammation promote RoI by regulating several molecular and cellular pathways. Hence, the search for ligands characterized by an adequate pharmacological profile and bioavailability, which may become widely used to promote endogenous RoI through FPR2 activation, appears advisable and may be a promising strategy for resolution pharmacology in the future.

## Abbreviations

Aβ: Amyloid β
AD: Alzheimer’s disease
Akt: Protein kinase B
ANXA1: Annexin A1
AP-1: Activator protein 1
ASA-COX2: Acetylated cyclooxygenase
ATL: Aspirin-triggered lipoxin
AT-LXA4, AT-LXB4: Aspirin-triggered lipoxins
cAMP: Cyclic adenosine monophosphate
CNS: Central nervous system
COX-2: Cyclooxygenase 2
DHA: Docosahexaenoic acid
DAMP: Damage-associated molecular patterns
EPA: Eicosapentaenoic acid
ERK 1/2: Extracellular signal-regulated kinases
fMLF: N-formyl-methionyl-leucylphenylalanine
FPR: Formyl peptide receptor
GPCR: G-protein-coupled receptors
GPR32: G-protein-coupled receptor 32
IFN-γ: Interferon-γ
IL: Interleukin
LOX: Lipoxygenase
LPS: Lipopolysaccharide
LXA4: Lipoxin A4
MAPK: Mitogen-activated protein kinase
MARCO: Macrophage scavenger receptor
NF-κB: Nuclear factor-κB
Nrf2: Nuclear factor erythroid 2-related factor 2
P38MAPK: P38 mitogen-activated protein kinases
PAMP: Pathogen-associated molecular patterns
PPARγ: Peroxisome proliferator-activated receptor gamma
PI3K: Phosphoinositide 3-kinase
PKC: Protein kinase C
PLC: Phospholipase C
PrP^c^: Cellular prion protein
PRR: Pathogen recognition receptors
PUFA: Polyunsaturated fatty acid
RoI: Resolution of inflammation
ROS: Reactive oxygen species
RvD1: Resolvin D1
SAA: Serum amyloid A
SOCS: Cytokine signaling suppressors
SPMs: Specialized pro-resolving mediators
TLR4: Toll-like receptor 4
TNF-α: Tumor necrosis factor α
